# Vascular endothelial growth factor receptor and coreceptor expression in human acute respiratory distress syndrome^☆^^☆☆^

**DOI:** 10.1016/j.jcrc.2008.04.003

**Published:** 2009-06

**Authors:** Andrew R.L. Medford, Nassif B.N. Ibrahim, Ann B. Millar

**Affiliations:** a Lung Research Group, Department of Clinical Science at North Bristol, University of Bristol, Southmead Hospital, Westbury-on-Trym, Bristol BS10 5NB, United Kingdom; b Department of Pathology, Frenchay Hospital, Frenchay, Bristol BS16 1LE, United Kingdom

**Keywords:** Acute respiratory distress syndrome, Vascular endothelial growth factor, Neuropilin, Receptors

## Abstract

**Background:**

Acute respiratory distress syndrome (ARDS) is characterized by the development of noncardiogenic pulmonary edema, which has been related to the bioactivity of vascular endothelial growth factor (VEGF). Vascular endothelial growth factor receptors and coreceptors regulate this bioactivity. We hypothesized VEGF receptors 1 and 2 (VEGFR1, VEGFR2) and coreceptor neuropilin-1 (NRP-1) would be expressed in human lung tissue with a significant change in expression in ARDS lung.

**Methods:**

Archival “normal” (no lung pathology and non-ARDS), “early” (within 48 hours), and “later” (after day 7) ARDS lung-tissue sections (n = 5) were immunostained for VEGFR1, VEGFR2, and NRP-1 from human subjects (n = 4). Staining was assessed densitometrically using Histometrix software.

**Results:**

VEGFR1, VEGFR2, and NRP-1 were expressed on both sides of the alveolar-capillary membrane in both normal and ARDS human lung tissue. In later ARDS, there was a significant up-regulation of VEGFR1 and VEGFR2 versus normal and early ARDS (*P* < .0001). Neuropilin-1 was down-regulated in early ARDS versus normal lung (*P* < .05), with normalization in later ARDS (*P* < .001).

**Conclusion:**

Differential temporal VEGFR1, VEGFR2, and NRP-1 up-regulation occurs in human ARDS, providing evidence of further functional regulation of VEGF bioactivity via VEGFR2 consistent with a protective role for VEGF in lung injury recovery. The mechanisms behind these observations remain to be clarified.

## 1 Introduction

Acute respiratory distress syndrome (ARDS) is the most extreme form of acute lung injury. It is characterized by noncardiogenic pulmonary edema, neutrophilic alveolitis, and the development of potentially reversible fibrosis, but its pathogenesis still remains incompletely explained [1]. Vascular endothelial growth factor (VEGF) is a potent angiogenic factor of critical importance in vascular development [2]. Its other characteristics as a potent permeability and mitogenic factor on vascular endothelium have led to investigating its possible role in ARDS [3-6], which continues to have an unacceptable morbidity and mortality of at least 35% despite improvements in management of sepsis and ventilatory support [7].

Observational data show plasma soluble VEGF levels rise and intrapulmonary levels fall in the early stages of lung injury with normalization of both during recovery [3,4,8]. Vascular endothelial growth factor protein is compartmentalized to high levels in normal human epithelial lining fluid, and human type 2 epithelial (ATII) cells express significant amounts of VEGF protein in vitro [9,10].

Vascular endothelial growth factor exerts its biological effect on vascular endothelium through specific receptors, VEGFR1 and VEGFR2. They have 7 immunoglobulin-like domains with specific functions, a single transmembrane region, and a consensus tyrosine kinase sequence interrupted by a kinase-insert domain [11]. Vascular endothelial growth factor also acts indirectly via the coreceptor neuropilin-1 (NRP-1), which augments VEGFR2 signaling activity but lacks tyrosine kinase activity itself and is therefore only able to act indirectly via effects on VEGFR2 activity [12,13].

Vascular endothelial growth factor receptor 2 is thought to be the main signaling receptor [14], and VEGFR1 has been speculated to function as a decoy receptor [15]. Both of these receptors were initially thought to be largely confined to the vascular bed (on endothelial cells), but studies in animal and developing human lung confirm expression in lung tissue on activated macrophages and respiratory epithelial cells [16-18]. This implies that VEGF can exert its biological effects in the alveolar compartment as well as the vascular bed and is the subject of ongoing study.

As well as functionally regulating VEGF bioactivity, VEGF receptors are themselves subject to functional regulation by oxygen tension and VEGF itself, at least in the vascular bed. Chronic hypoxia up-regulates VEGFR1 and VEGFR2 expression in vivo [19]. Vascular endothelial growth factor receptor 1 has a hypoxia-inducible factor 1 consensus sequence in its promoter region, whereas VEGFR2 does not and is thought to be up-regulated by posttranscriptional paracrine mechanisms [20]. Vascular endothelial growth factor activation of VEGFR2 increases VEGFR2 gene expression and cellular levels, and VEGF can also up-regulate VEGFR1 expression in endothelial cells [21,22].

One explanation for the observed reduction in soluble intrapulmonary VEGF levels in early ARDS would be an increased expression of VEGF receptors facilitating an increased number of VEGF binding sites. Other possibilities (which are not mutually exclusive) include an alteration in alternate splicing or soluble VEGF inhibitors which have been investigated elsewhere [23]. We therefore hypothesized that VEGFR1, VEGFR2, and NRP-1 would be expressed in the adult human lung alveolar compartment as well as the vascular bed with dynamic temporal changes in expression in ARDS consistent with a role in lung repair after injury.

## 2 Methods

### 2.1 Specimens

Archival normal and ARDS lung-tissue sections and paraffin blocks were obtained from Frenchay Pathology Department. The North Bristol NHS Trust Local Research Ethics Committee approved this study.

### 2.2 Immunohistochemistry

Normal, early, and late ARDS lung-tissue sections were obtained from human subjects (n = 4 for each group). Normal lung tissue implied that there was no lung involvement in the cause of death and no ARDS. Acute respiratory distress syndrome lung tissue was subdivided into “early” (within 48 hours of onset) and “late” (after day 7). Paraffinized 4-μm sections (n = 5 for each subject) were dewaxed in serial xylene (BDH Laboratory Supplies, Poole, UK), dehydrated in absolute ethanol (BDH Limited Laboratory Supplies), and pressure cooked in 0.01 M trisodium citrate (BDH Laboratory Supplies) buffer (pH 6) to facilitate antigen retrieval. Saponin (0.1%; Sigma-Aldrich, Dorset, UK) in phosphate-buffered saline (Oxoid, Basingstoke, UK), pH 7.3, was used as a wash buffer and antibody diluent. Endogenous peroxidase was blocked with 3% hydrogen peroxide (BDH Laboratory Supplies) in methanol (BDH Laboratory Supplies).

Sections were incubated in 2.5% horse blocking serum (Vectastain Universal Quick Kit; Vector Laboratories, Peterborough, UK) before avidin D and biotin blocking sera (Vector Laboratories). Rabbit polyclonal antibodies to VEGFR1, VEGFR2, and NRP-1 (Autogen Bioclear; UK Ltd, Wiltshire, UK) were used as primary antibodies. Isotypic rabbit immunoglobulin G (Vector Laboratories) at the same concentration was used as a negative control. A pan-specific biotinylated antibody, streptavidin-peroxidase complex with diaminobenzidine substrate (Vectastain Universal Quick Kit; Vector Laboratories) was added. Sections were counterstained in hematoxylin (BDH Laboratory Supplies) before serial washes in absolute ethanol and xylene before mounting with Distrene-80 Plasticizer Xylene (DPX) (BDH Laboratory Supplies). Image capture and quantitative densitometry were performed with Histometrix software (Kinetic Imaging Limited, Wirral, Merseyside, UK).

### 2.3 Statistical analysis

All statistical analyses were performed using GraphPad Prism version 4.0 software. Data in bar charts are plotted as mean and standard error. Quantitative immunostaining Histometrix pixel staining densities were normally distributed as assessed by the Ryan-Joiner test. Because of the necessity for multiple comparisons of the data, analysis of variance testing was followed by Bonferroni post hoc analysis. A *P* value less than .05 was considered significant.

## 3 Results

### 3.1 Immunohistochemistry

**Fig. 1:**
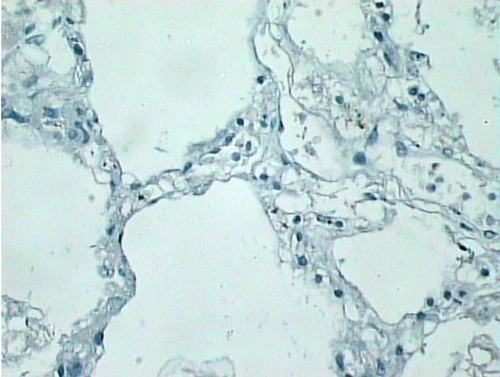
Isotypic control staining in early ARDS lung (original magnification, ×40).

**Fig. 2:**
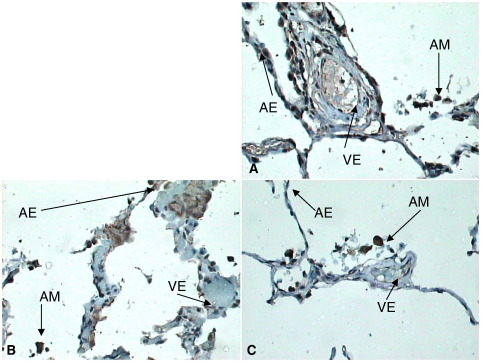
A–C, VEGFR2 (A), NRP-1 (B), and VEGFR1 (C) expression in normal lung (original magnification, ×40). Receptor expression on endothelium strong. Expression of all receptors noted on alveolar epithelium and macrophages. Relative VEGFR2 expression generally noted to be higher generally in normal lung. AM indicates alveolar macrophages; AE, alveolar epithelium; VE, vascular endothelium.

**Fig. 3:**
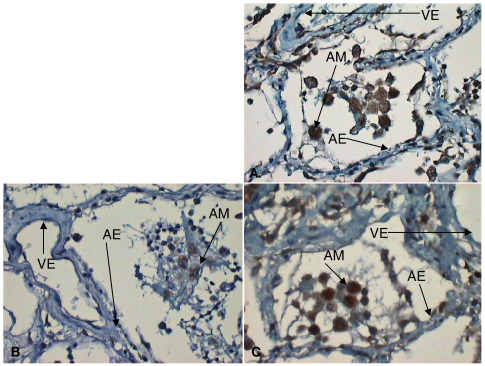
A–C, VEGFR2 (A), NRP-1 (B), and VEGFR1 (C) expression in early ARDS showing consistently reduced alveolar expression of VEGFR2 and NRP-1 (highlighted; original magnification, ×40). Endothelial and macrophage expression as before. AM indicates alveolar macrophages; AE, alveolar epithelium; VE: vascular endothelium.

**Fig. 4:**
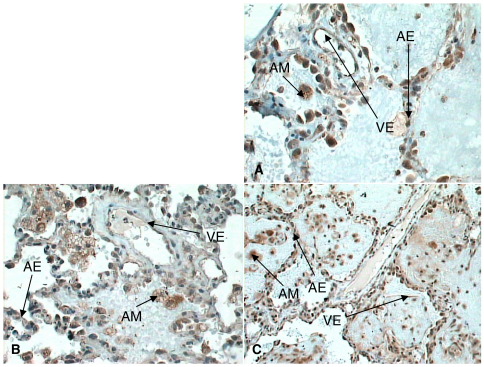
A–C, VEGFR2 (A), NRP-1 (B), and VEGFR1 (C) expression in later ARDS lung (original magnification, ×40). Increased expression for all receptors noted on alveolar epithelium, macrophages, and endothelium. AM indicates alveolar macrophages; AE, alveolar epithelium; VE, vascular endothelium.

Indirect immunohistochemistry revealed no evidence of nonspecific staining using isotypic negative control antibodies confirming that positive staining was significant (Fig. 1). In addition to expected vascular endothelial expression, VEGFR1, VEGFR2, and NRP-1 expression was noted on alveolar epithelium and macrophages (Figs. 2–4A–C).

On direct visualization (before densitometry), differential immunostaining was noted. In normal lung tissue, VEGFR2 staining was most intense (Fig. 2A). In early ARDS, a relative loss of alveolar epithelial expression of VEGFR1, VEGFR2, and NRP-1 especially the latter was noted (Fig. 3A–C). In later ARDS, there was marked up-regulation of expression of VEGFR1, VEGFR2, and NRP-1, although of less magnitude with the latter (Fig. 4A–C).

### 3.2 Staining densitometry

**Fig. 5:**
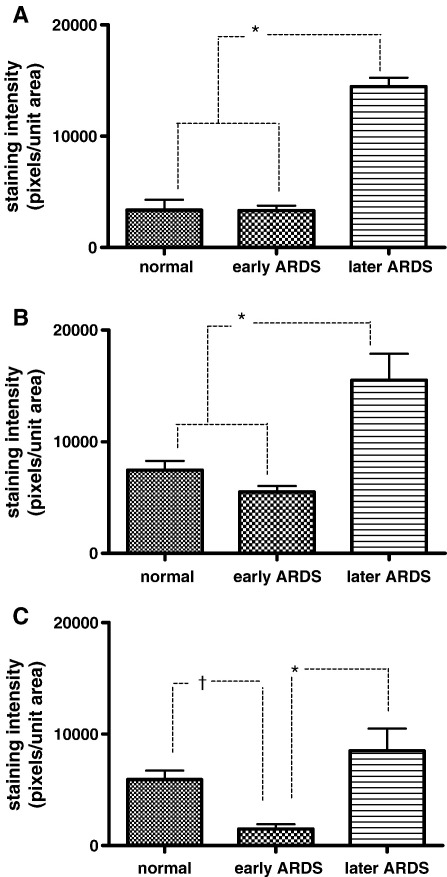
A–C, Graphs of quantitative immunostaining densities (plotted as pixels of staining per unit area) for VEGFR1 (A), VEGFR2 (B), and NRP-1 (C) in each disease state. All data are normally distributed and plotted as mean and standard error. A–B, *P* < .001 (Bonferroni) for normal versus late and early versus late (highlighted*); otherwise, other comparisons are not significant. C, *P* < .001 (Bonferroni) for early versus late (highlighted*); *P* < .05 (Bonferroni) for normal versus early (highlighted^†^); otherwise, other comparisons are not significant.

Histometrix densitometric analysis supported the visual observations noticed in immunostaining and is presented graphically in Fig. 5A–C. Differential time-dependent changes in VEGFR1, VEGFR2, and NRP-1 expression were noted. Vascular endothelial growth factor receptors 1 and 2 expression was significantly up-regulated in the later stages of ARDS (*P* < .001, Bonferroni) versus normal and early ARDS lung (Fig. 5A–B).

In contrast to VEGFR1 and VEGFR2, NRP-1 expression was down-regulated in early ARDS lung (*P* < .05, Bonferroni) versus normal lung with significant up-regulation in later ARDS (*P* < .001, Bonferroni) versus early ARDS (Fig. 5C). Moreover, unlike the other receptors, later ARDS NRP-1 expression did not significantly differ from normal lung (Fig. 5C).

## 4 Discussion

Vascular endothelial growth factor is compartmentalized to high levels in normal human epithelial lining fluid, 500 times higher than plasma levels [9]. In health, significant angiogenesis does not occur in the lung, implying that this VEGF reservoir has another function. Observational data suggest that VEGF may have a role in recovery from lung injury with temporal alterations in plasma and intrapulmonary VEGF levels correlating with injury and normalization in recovery [3,4].

Potential explanations for this apparent reduction in intrapulmonary VEGF levels in early ARDS are manifold and not mutually exclusive. Vascular endothelial growth factor bioactivity is subject to functional regulation including by VEGF receptors and coreceptors. Vascular endothelial growth factor receptors are themselves subject to functional regulation by oxygen tension and VEGF itself. One important explanation for the VEGF reduction in early ARDS is by up-regulated specific VEGF receptor expression.

We therefore assessed VEGF-specific receptor (VEGFR1, VEGFR2) and coreceptor NRP-1 expression by immunohistochemistry in archival normal and ARDS lung tissue, anticipating that functional regulation might occur. We found VEGFR1, VEGFR2, and NRP-1 expression on alveolar macrophages and epithelium (consistent with data from animal and developing lung studies) [16-18] in addition to their known expression on vascular endothelium. We have demonstrated differential up-regulation of VEGFR1, VEGFR2, and NRP-1 expression in human ARDS. Neuropilin-1 was uniquely down-regulated in early ARDS with up-regulation in later ARDS. VEGFR1 and VEGFR2 were up-regulated in later ARDS. Our data are consistent with a reduced VEGFR2 (due to reduced NRP-1) in early ARDS with an up-regulated VEGFR2 signal (via up-regulation of VEGFR2 and NRP-1) in later ARDS.

As VEGFR2 is the main VEGF signaling receptor, these data are consistent with a role of VEGF in repair after lung injury. We speculate that the up-regulation of decoy receptor VEGFR1 in later ARDS may serve as a functional and spatial regulator of VEGF activity via VEGFR2. Although statistical comparison between the different receptor staining was not possible (as different antibody affinities would not have been identical), the staining intensities of both VEGFR2 (7450 pixels/unit area) and NRP-1 (5920 pixels/unit area) were noted to be higher than for VEGFR1 (3358 pixels/unit area) in normal lung, suggesting predominant VEGFR2 signaling in both the normal lung as well as in the injured (but recovering) lung. These observations are consistent with an autocrine VEGF role in the lung on alveolar epithelium in addition to its established paracrine action on endothelium secreted from the epithelium. Such an autocrine mechanism has been already described in epithelial cells in the kidney but not yet in the lung [24].

Given the lack of previous studies and the difficulty in obtaining human ARDS lung tissue, our study adds significant and novel data to current knowledge. We have looked at protein level changes with time in human ARDS and quantified our immunostaining with biologically plausible results.

We acknowledge the limitations of this study. Immunohistochemistry is primarily a localization technique, although use of Histometrix in our study facilitated quantification. Surgical lung biopsy from living ARDS patients would have been preferred, but this is seldom performed due to the rapidity of onset of ARDS, lack of thoracic surgeons on site, and consent issues. We circumvented this problem using necropsy lung tissue, although we cannot exclude selection bias for a more severe spectrum of disease. Because contemporaneous bronchoalveolar lavage fluid was unavailable from the ARDS subjects, we were unable to measure contemporaneous levels of soluble VEGF receptors which would be an important addition in future studies to complement our work.

Existing human VEGF receptor regulation studies in ARDS conflict. In agreement with our immunohistochemical data, Lassus et al [25] demonstrated persistent expression of VEGFR1 on vascular endothelial, bronchial epithelial, and ATII cells in developing lungs with bronchopulmonary dysplasia throughout the fetal and neonatal period. No densitometry assessments were made. Perkins et al [23] detected the presence of significant quantities of intrapulmonary soluble VEGFR1 protein in early ARDS but did not study VEGFR1, VEGFR2, or NRP-1. Conversely, Tsokos et al [26] detected reduced VEGFR2 mRNA by real-time polymerase chain reaction in human necropsy lung tissue from septic patients. However, protein was not assessed, nor was VEGFR1 or NRP-1; the patients did not strictly conform to an ARDS phenotype and were assessed at varying time points (4-28 days).

In animals, data also conflict. The strongest evidence comes from intervention studies. Chronic VEGFR2 blockade in rats leads to alveolar apoptosis and emphysema [27], suggesting a role in recovery from lung injury and a possible survival function for VEGF via VEGFR2 on alveolar epithelium. In contrast to our data, many studies demonstrate a down-regulation in VEGF receptor expression after injury in the early stages. Ito et al [28] observed a reduction in VEGFR1 and VEGFR2 mRNA expression 24 to 72 hours after LPS-induced murine lung injury in both young and old mice. Time points were earlier and protein was not measured. Mura et al [29] noted a reduction in lung VEGFR1 (but not VEGFR2) expression in an ischemic/reperfusion-induced murine mode of lung injury. This study also confirmed VEGFR1 and VEGFR2 expression on both sides of the alveolar-capillary membrane but with discordant features: notably, a reduction in alveolar epithelial, alveolar macrophage, and interstitial VEGFR1 and a redistribution of VEGFR2 positive cells into the interstitium and ATII cells. Time points were again earlier (4 hours postinjury). Klekamp et al [18] noted a decrease in VEGFR1 and VEGFR2 mRNA expression at 48 hours after hyperoxic lung injury in rats. Protein was not assessed. Tambunting et al [30] detected reduced mRNA expression of VEGFR1, VEGFR2, and NRP-1, whereas Maniscalco et al [31] observed reduced VEGFR1 mRNA expression only (NRP-1 expression was not assessed in the latter) both in baboon models of bronchopulmonary dysplasia. Differences in methodology (ribonuclease protection assay vs quantitative real-time polymerase chain reaction) might account for these findings.

Conversely, other animal studies suggest a pathological role for VEGF in lung injury. Gurkan et al [32] noted an increase in lung VEGFR2 protein after an acid-induced murine lung injury model following high-tidal volume ventilation, attenuated by a protective ventilatory strategy. Time points were earlier (4 hours). Kazi et al [33] noted a similar up-regulation of lung VEGFR2 mRNA and protein expression for 24 hours in an ischemic model of murine lung injury. Time points were earlier, and VEGFR1 and NRP-1 were not studied.

What are the implications of these findings? Existing data conflict due to differing species, time points after injury, phenotyping, methodologies, and the receptors studied. Our data provide a potential mechanism for further functional regulation of VEGF bioactivity with a reduced VEGFR2 signal in early injury and enhanced VEGFR2 signal in later ARDS, providing further support for an autocrine protective role for VEGF in the injured lung acting via VEGFR2. Interventional studies suggest that these observations are not epiphenomena.

Local VEGF delivery may be a potential new therapy for acute lung injury, but further studies are needed to clarify the mechanisms and functional consequences of these observations. These will include functional in vivo assessments of VEGF receptor expression, receptor signaling studies, and further VEGF receptor knockout studies in animal models of lung injury.
